# Obesity paradox among patients undergoing total knee arthroplasty: a retrospective cohort study

**DOI:** 10.1186/s12893-022-01806-6

**Published:** 2022-11-02

**Authors:** Lulu Ma, Xuerong Yu, Xisheng Weng, Jin Lin, Wenwei Qian, Yuguang Huang

**Affiliations:** 1 Department of Anesthesiology, Peking Union Medical College Hospital, 100730 Beijing, China; 2 Department of Orthopedics, Peking Union Medical College Hospital, 100730 Beijing, China

**Keywords:** Obesity paradox, Total knee arthroplasty, Postoperative complications

## Abstract

**Background:**

Obesity has been recognized as the risk factor for postoperative complication for surgical patients. However, recent studies have showed protective effect of obesity in surgical and non-surgical patients. Our study is to examine the association of body mass index(BMI) with early postoperative complications in patients undergoing total knee arthroplasty.

**Materials and methods:**

All patients who had primary total knee arthroplasty between January 2014 and December 2019 were included. Medical records were retrospectively reviewed and BMI was categorized as underweight(BMI < 18.5), normal weight(18.5 < BMI < 24.9), overweight I(25 < BMI < 27.4), overweight II(27.5 < BMI < 29.9), obese I(30 < BMI < 34.9) and obese II(BMI ≥ 35). The association between BMI and occurrence of early postoperative complications was examined and logistic regression was used to calculate relationship between BMI and early postoperative complications.

**Results:**

A total of 2969 patients were included in our analysis. The overall complication rate in patients undergoing total knee arthroplasty was 14.8%, with the highest complication being 22.2% in the underweight group, the second highest in the normal weight group(17.5%), the lowest in the overweight I(13.8%) and obese I(12.0%) group and then higher again in obese II group(16.7%). In multivariable analyses, overweight I (OR 0.737, 95% CI 0.559–0.972, *P* = 0.031) and obese I (OR 0.631, 95% CI 0.449–0.885, *P* = 0.008) were associated with lower risk of early postoperative complications after total knee arthroplasty.

**Conclusion:**

In this retrospective study, overweight and obese patients had a lower risk of early postoperative complications after total knee arthroplasty. Further studies are necessary to confirm and investigate the mechanism of obesity paradox in this surgical population.

**Trial registration:**

This study had been registrated in www.chictr.org.cn on 25/10/2021 and the registration ID was ChiCTR2100052408.

## Introduction

Obesity has been considered as a public health problem both in developed [1, 2] and developing countries [3, 4]. It has been proven that obesity is associated with comorbidities [5], which include hypertension, diabetes and coronary artery disease, and postoperative complications [6]. However, recent research had demonstrated the protective effect of obesity in both surgical [1, 7, 8] and non-surgical patients [9–12]. This phenomenon has been called “obesity paradox”, which means better outcome in patients with higher BMI.

Since obesity increases the risk of osteoarthritis, the prevalence of obesity is related to the growing demands of joint arthroplasty [13]. The role of obesity in outcome of joint arthroplasty was still controversary. Although the negative effects of obesity on the outcome after total knee arthroplasty (TKA) and/or hip arthroplasty(THA) [14, 15] had been reported, some studies demonstrated the obesity paradox applied to early postoperative complications after hip or knee surgeries [16, 17].

The purpose of our study was to evaluate the incidence of early postoperative complications stratified by body mass index.

## Materials and methods

We conducted a retrospective review of the electronic medical records of patients who underwent primary TKA (unilateral TKA or simultaneous bilateral TKA) at our institution from January 2014 to December 2019. This study had been registrated in www.chictr.org.cn on 25/10/ 2021 and the registration ID was ChiCTR2100052408. The study was approved by Institutional Review Board (IRB) review of Peking Union Medical College Hospital and the need for informed consent was waived by institutional review board of Peking Union Medical College Hospital due to the retrospective nature of our study.

The exclusion criteria included: patients who had TKA and THA simultaneously, patients who had unicompartmental knee arthroplasty or revision surgery and patients who had incomplete data of weight and/or height. BMI was calculated by using the standard formula of weight in kilograms divided by the square of height in meters. BMI was categorized into 6 groups [8]: underweight(< 18.5 kg/m^2^), normal weight (18.5–24.9 kg/m^2^), overweight I (25-27.4 kg/m^2^), overweight II(27.5–29.9 kg/m^2^) and obese I (30-34.9 kg/m^2^) and obese II (≥ 35 kg/m^2^).

Electronic medical records were reviewed and demographic data were collected, which included age, sex, BMI, American society of Anesthesiologists physical status(ASA class), New York Heart Association(NYHA) classification, history of hypertension, diabetes mellitus (DM), coronary artery disease (CAD), chronic obstructive pulmonary disease(COPD) and cerebrovascular disease or accident(CVD). Intraoperative and postoperative data were also collected, which included the duration of anesthesia, tourniquet and operation, type of anesthesia, type of surgery, admission to intensive care unit (ICU), the length of hospital stay and postoperative complications. Laboratory results, including baseline hemoglobin were also collected for analysis.

Anemia was defined according to WHO scientific Group report, with a cutoff value of hemoglobin < 130 g/L for adult men and < 120 g/L for adult nonpregnant women. Early postoperative complications were defined as complications occurred during hospital stay. The severity of postoperative complications were defined according to Clavien-Dindo classification system [18]. For patients who had more than 1 complication, the grade of the most severe complication was recognized as the severity of complications. Complications of grade ≥ 3 were defined as severe complications [19].

Categorical variables were described as number (percentage) and chi-square test was used to compare difference across BMI categories. Continuous data were presented as means ± standardized deviation (SD) or median [25th percentile, 75th percentile interquartile range(IQR)], analysis of variance (ANOVA) or Kruskal-Wallis test was used to compare across BMI classes. Logistic regression analysis was performed to calculate the adjusted odds ratios for the occurrence of complications in the BMI categories with the normal weight group (BMI 18.5–24.9 kg/m^2^) as reference. All reported *P* value were 2 sided, and a *P* value < 0.05 was considered statistically significant. Standardized statistical software (SPSS 23, CHICAGO, IL) was used for statistical analysis.

## Results

A total of 2969 patients were included in our analysis. 27 patients (0.9%) were underweight, 905 (30.5%) were normal weight,1478 (49.8%) were overweight and 559 patients (18.8%) were obese. Demographic characteristics were presented in Table 1. Overweight and obese patients were more likely to be female and had higher ASA class. Patients with higher BMI were more prone to have hypertension(*P* < 0.001), CAD(*P* = 0.046) and DM(*P* = 0.008). There was no significant difference in CVD(*P* = 0.671) and COPD(*P* = 0.259) among different BMI groups. Patients with lower BMI were associated with lower preoperative hemoglobin level(*P* = 0.042) and higher proportion of preoperative anemia(*P* < 0.001).

**Table 1:** Comparison of patient characteristics in different BMI groups

	Underweight	Normal weight	Overweight		Obese		***P***
	(n = 27)	(n = 905)	I(n = 863)	II(n = 615)	I(n = 500)	II(n = 59)	
Age, year old	51.5 ± 19.7	66.8 ± 10.6	66.6 ± 7.8	66.8 ± 7.6	66.0 ± 7.9	65.4 ± 7.0	< 0.001
Old than 65 years old	8(29.6%)	544(60.1%)	500(57.9%)	343(55.8%)	259(51.8%)	28(47.5%)	0.003
Female	16(59.3%)	686(75.8%)	710(82.3%)	497(80.8%)	436(87.2%)	54(91.5%)	< 0.001
ASA ≥ 3	0	55(6.1%)	81(9.4%)	39(6.3%)	45(9.0%)	5(8.5%)	0.035
Diagnosis							< 0.001
OA	9(33.3%)	801(88.5)	816(94.6%)	593(96.4%)	480(96.0%)	58(98.3%)	
Diagnosis other than OA	18(66.7%)	104(11.5%)	47(5.4%)	22(3.6%)	20(4.0%)	1(1.7%)	
Comorbidity							
Hypertension	0	411(45.4%)	486(56.3%)	392(63.7%)	350(70.0%)	42(71.2%)	< 0.001
CAD	2(7.4%)	87(9.6%)	102(11.8%)	77(12.5%)	77(15.4%)	8(13.6%)	0.046
CVD	2(7.4%)	47(5.2%)	52(6.0%)	38(6.2%)	25(5.0%)	1(1.7%)	0.671
COPD	1(3.7%)	17(1.9%)	11(1.3%)	10(1.6%)	15(3.0%)	2(3.4%)	0.259
Diabetes mellitus	0	149(16.5%)	176(20.4%)	123(20%)	91(18.2%)	17(28.8%)	0.008
Hemoglobin(g/l)	122 ± 14	131 ± 14	134 ± 41	133 ± 13	132 ± 12	132 + 10	0.042
Preoperative anemia	13(48.1%)	191(21.1%)	113(13.1%)	75(12.2%)	77(15.4%)	5(8.5%)	< 0.001

**Fig. 1:**
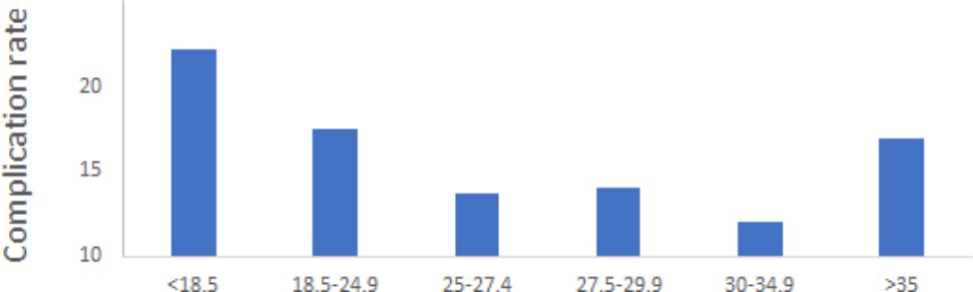
The incidence of postoperative complication rate in different BMI groups

Patients in overweight and obese groups had longer length of operation (*P* = 0.001)and tourniquet(*P* < 0.001). And patients with higher BMI were more likely to had simultaneous bilateral total knee arthroplasty(*P* = 0.010)(See Table 2). The overall rate of all complications and severe complications in patients undergoing primary total knee arthroplasty was 14.8% and 2.6%, respectively. There was no significant difference in the incidence of all complications(P = 0.056) and severe complications(*P* = 0.425) among different BMI groups(See Table 3). The distribution of all complications demonstrated a J-shaped distribution, with lowest incidence of in the obese I group(See Fig. 1).Although there was no statistical significance in length of stay in hospital among different BMI groups, patients in underweight group had the longest LOS (*P* = 0.163) and highest rate of admission to ICU(*P* = 0.510).

**Table 2:** Comparisons of intraoperative parameters among different BMI groups

	Underweight	Normal weight	Overweight		Obese		***P***
	(n = 27)	(n = 905)	I(n = 863)	II(n = 615)	I(n = 500)	II(n = 59)	
Duration of surgery(minutes)	130 ± 55	119 ± 54	124 ± 54	125 ± 55	130 ± 57	142 ± 65	0.001
Duration of tourniquet(minutes)	94 ± 46	98 ± 48	106 ± 49	106 ± 49	111 ± 51	109 ± 52	< 0.001
Anesthesia method							0.120
General anesthesia	24(88.9%)	843(93.1%)	818(94.8%)	584(95.0%)	482(96.4%)	56(94.9%)	
Intrathecal anesthesia	3(11.1%)	62(6.9%)	45(5.2%)	31(5.0%)	18(3.6%)	3(5.1%)	
The type of surgery							0.010
Unilateral TKA	19(70.4%)	644(71.2%)	555(64.3%)	402(65.4%)	314(62.8%)	36(61%)	
Bilateral TKA	8(29.6%)	261(28.8%)	308(35.7%)	213(34.6%)	186(37.2%)	23(39.0%)	

**Table 3:** Comparisons of outcomes of patients in different BMI groups

	Underweight	Normal weight	Overweight		Obese			***p***
	(n = 27)	(n = 905)	I(n = 863)	II(n = 615)	I (n = 500)		II (n = 59)	
All-type complications	6 (22.2%)	158 (17.5%)	118(13.7%)	86(14.0%)	60(12.0%)		10(16.9%)	0.056
Severe complications	0	27(3.0%)	16(1.9%)	15(2.4%)	11(2.2%)		3(5.1%)	0.425
Subtype complications								
Venous thromboembolism	1(3.7%)	26(2.9%)	23(2.7%)	17(2.8%)	11(2.2%)		0	0.801
Transfusion	4(14.8%)	112(12.4%)	77(8.9%)	62(10.1%)	44(8.8%)	9(15.3%)		0.097
Wound complication	1(3.7%)	19(2.1%)	9(1.0%)	14(2.3%)	6(1.3%)		0	0.155
Pulmonary complication	0	1(0.1%)	6(0.7%)	2(0.3%)	3(0.6%)		2(3.4%)	0.012
Cardiac complication	0	9(1.0%)	10(1.2%)	5(0.8%)	3(0.6%)		1(1.7%)	0.778
Urinary tract infection	0	8(0.9%)	3(0.3%)	2(0.3%)	1(0.2%)		1(1.7%)	0.238
Neurologic complication	0	4(0.4%)	0	4(0.7%)	1(0.2%)		2(3.4%)	0.001
Length of hospital stay(day)	8[6, 9]	7[4, 9]	7[4, 9]	6[4, 9]	6[4, 8]		6[5, 11]	0.163
Admission to ICU	1(2.7%)	12(1.3%)	7(0.8%)	4(0.7%)	6(1.2%)		1(1.7%)	0.510

Age, sex, ASA class, diagnosis, hypertension, CAD, DM, preoperative anemia,the type of operation, the duration of the operation and tourniquet, and the 6 BMI classes were entered into the logistic regression model.Of these variables, age(OR 1.023, 95%CI 1.010–1.036, *P* = 0.001), ASA class(OR 1.315, 95%CI 1.034–1.672, *P* = 0.026), diagnosis(OR 1.069, 95%CI 1.019–1.121, *P* = 0.006), preoperative anemia(OR 2.316, 95%CI 1.792–2.994, *P* < 0.001), the type of operation (OR 1.938, 95%CI 1.358–2.769, *P* < 0.001)and the duration of the operation(OR 1.009, 95%CI 1.006–1.012, *P* < 0.001) were risk factors of postoperative complications after total knee arthroplasty( See Table 4). Overweight I (OR 0.737, 95%CI 0.559–0.972, *P* = 0.031) and obese I(OR 0.631, 95%CI 0.449–0.885, *P* = 0.008) were the risk factors associated with less early postoperative complications after total knee arthroplasty( See Table 4).

**Table 4:** Logistic regression of analysis for the incidence of early postoperative complications

Variables	OR	95% CI			P
		lower	upper		
Age	1.023	1.010		1.036	0.001
Sex	0.851	0.643		1.127	0.260
ASA class Diagnosis	1.315 1.069	1.034 1.019		1.672 1.121	0.026 0.006
Hypertension	0.872	0.691		1.100	0.149
CAD	1.296	0.940		1.787	0.113
DM	1.215	0.924		1.596	0.163
Preoperative anemia	2.316	1.792		2.994	< 0.001
The type of operation	1.938	1.358		2.769	< 0.001
The duration of the operation	1.009	1.006		1.012	< 0.001
The duration of the tourniquet	0.998	0.996		1.001	0.129
Undereweight	1.079	0.401		2.901	0.880
Overweight I	0.737	0.559		0.972	0.031
Overweight II	0.990	0.738		1.328	0.947
Obese I	0.631	0.449		0.885	0.008
Obese II	0.747	0.342		1.632	0.464

## Discussion

In this retrospective study, we demonstrated a J-shaped pattern between BMI and early postoperative complications. Although not statistically significant, risk of early postoperative complications tended to be higher in underweight group when compared to normal weight group. Overweight I and obese I were the predictive risk factors of less early postoperative complications. Our results did support obesity paradox in patients undergoing total knee arthroplasty.

Previous studies had demonstrated the non-linear relationship between BMI and outcome in patients undergoing total knee or hip arthroplasty[16, 20, 21]. However there results were not consistent and few studies focused on obesity paradox in patients undergoing total knee arthroplasty. George had demonstrated the U-shaped relationship between BMI and readmission & reoperation after total knee arthroplasty [21],however this study did not include underweight patients. Other studies showed the strong relationship between BMI and postoperative complications[22, 23] after total knee arthroplasty. Patients in the underweight, normal and morbidly obese groups had the highest incidence of early postoperative complications after total hip arthroplasty [16], but Zhang demonstrated that selection bias may contribute obesity paradox since patients who were morbidly obese did not have a reduced risk of death in 30 days after urgent hip surgery [24]. In our study, we did find the highest complication rate in the group of underweight (22.2%), while patients in the group of obese I had the lowest complication rate. However, patients in underweight or obese II group only represented 0.9% and 2.0% of the whole study population, and the result should be interpreted with caution.

The protective effect of obesity in certain chronic disease and postoperative period for surgical patients had been reported previously. The exact underlying mechanisms were not clear, and several possible mechanisms had been suggested. First, lipoproteins may have protective effects against inflammatory mediators and endotoxins [25], which protect patients from the inflammatory reaction associated with surgery. Second, patients with low BMI are at a high risk of malnutrition and the nutrition status is associated with poor postoperative outcome [26–29]. Third, obesity paradox also reflects that BMI may not be the best indicator of obesity and the best cutoff point of BMI has not been determined [21, 30]. Besides these, patients in overweight or obese groups usually get more attention in the perioperative period. Preoperative comprehensive screening and optimization of cardiopulmonary function may lead to decreased complications after operation.

Our study had several limitations. First, this is a retrospective study in a single center and selection bias could not be avoided. Second, we only selected BMI as the category of obesity, and the waist-to-hip ratio or cholesterol level were not considered as the markers of obesity. Third, we only explore the association between BMI and in-hospital complications. The relationship between BMI and the long-term complications was not determined in our study.

In conclusion, our study suggested that overweight and obese patients are at lower risk of postoperative complications. Further research is necessary to determine the mechanism of the protective effect of weight and whether obesity paradox is a result of weight itself or due to other related effects.

## Data Availability

The datasets used during the current study are not publicly available due to local regulations, but are available from the corresponding author on reasonable request.
